# Association between negatively perceived parenting attitudes and dissociation: a cross-sectional study on the general population in Japan

**DOI:** 10.3389/fpsyg.2023.1235447

**Published:** 2023-08-17

**Authors:** Baihui Wang, Toshihide Kuroki

**Affiliations:** ^1^Faculty of Life Sciences, Center for Medical Education and Research, Kumamoto University, Kumamoto, Japan; ^2^Department of Clinical Psychology, Kyushu University Graduate School of Human-Environment Studies, Fukuoka, Japan

**Keywords:** dissociative experiences scale, negative perceived parenting, overprotection, attachment, general population

## Abstract

**Objectives:**

Many studies have reported that early traumatic experiences, mainly abuse, are associated with forming dangerous attachments and a contributing factor to dissociation. On the other hand, other studies have investigated the association of non-abusive nurturing and attachment styles with dissociation. The aim of this study is to determine the frequency of dissociative experiences in the general Japanese population and investigate the effects of “overprotection” and “lack of care” as nurturing styles and “abandonment anxiety” and “avoidance of intimacy” as attachment styles on dissociation.

**Methods:**

A total of 1,042 residents aged 18 to 69 years were administered with the Dissociative Experiences Scale (DES), the Japanese version of the WHO-5 Well-Being Index (WHO-5-J), the Parental Bonding Instrument (PBI), and the Experiences in Close Relationships Inventory-the-generalized-other-version (ECR-GO). Hierarchical multiple regression analyses on the effects of “overprotection” and “care” as nurturing attitudes “abandonment anxiety” and “avoidance of intimacy” as attachment styles on dissociation (DES-NDI and DES-T) were conducted.

**Results:**

Based on the findings of this study, “care” and “overprotection” as nurturing attitudes were shown to be a contributing factor to dissociation (DES-NDI and DES-T). “Avoidance of intimacy” as an attachment style was shown to contribute to pathological dissociation. On the other hand, the influence of attachment style on the relationship between nurturing style and dissociation was not determined.

**Discussion:**

This study provided essential data on the distribution of dissociative experiences in the general Japanese population. It was indicated that nurturing style, particularly overprotection, may be linked to nonfunctional stress coping and interpersonal anxiety and could be a contributing factor to mental disorders, including dissociation. Furthermore, considering that the effect of nurturing styles on dissociation does not vary with attachment styles, the effect of nurturing styles on dissociation may be more profound.

## 1. Introduction

Dissociation is the failure of thoughts, feelings, and experiences to integrate typically into streams of consciousness and memory (Bernstein and Putnam, 1986). This phenomenon has been characterized by mild immersive experiences to pathological conditions, such as dissociative disorders. Dissociative symptoms have been shown to coexist with a variety of psychiatric disorders, such as schizophrenia, eating disorders, panic disorders, mood disorders, and obsessive–compulsive disorders (Lyssenko et al., 2018). This is relevant to the clinical issue that dissociative symptoms may be underestimated in psychiatric conditions. An and Kaneda (1995) reported that, in Japan, it took an average of 4.6 years from the first visit to effectively diagnose dissociative identity disorder. For early detection and intervention of dissociation, the need has arisen to elucidate the factors involved in its formation in both clinical and non-clinical samples.

Kate et al. (2020) conducted a meta-analysis of 98 studies and reported that dissociation is widely experienced in general population, with an estimated prevalence of approximately 10%. In recent years, many studies on dissociation in Asian countries have focused on the development of scales for identifying pathological dissociation (Fung et al., 2018), traumatic experiences affecting pathological dissociation, and environmental risk factors for dissociation (Chu and Deprince, 2006; Xiao et al., 2006). In highlight of the significance of dissociation in the mental health field, previous studies indicate that high levels of dissociation are associated with adverse experiences, such as traumatic events, neglect, parental dysfunction, and chronic stress. Severe dissociation affects serious mental health struggles; complex emotional, social, and physical health disorders; functional impairment; and poor quality of life (Kate et al., 2023). Increased dissociative experiences are also associated with self-injury (Gratz et al., 2002; Anamizu and Tanaka, 2010) and aggressive behaviors (Tolmunen et al., 2010).

### 1.1. Dissociation and traumatic experiences

Dissociation is a defense mechanism against intolerable traumatic experiences, and many studies have suggested its association with trauma. In particular, childhood traumatic experiences are involved in developing dissociation in adulthood (Briere et al., 2008; Cloitre et al., 2009). Porges (2011) explained the relationship between childhood interpersonal traumatic experiences and dissociation in terms of neurophysiology based on his polyvagal theory. The autonomic nervous system is conventionally thought of as having two parts: the sympathetic nervous system and the parasympathetic nervous system. However, the polyvagal theory argues that the parasympathetic nervous system has a ventral vagus nerve in addition to the dorsal vagus nerve. The development of this ventral vagus coincides with the period of attachment formation, which is inhibited by the inappropriate functioning of the caregiver. In this situation, learning stress coping mechanisms (e.g., interacting with others produces calmness) becomes inadequate, and emotional self-regulation using social interactions becomes difficult. Especially in stressful solid situations, such as trauma, he suggested that dissociation is triggered as a shutdown (freeze state) since the dorsal vagal system tends to suppress the excessive activation of the sympathetic defense mechanisms (fight-or-flight) (Levine, 2010). Thus, inappropriate functioning of the caregiver has been shown to be closely related to the onset of dissociation. This finding is corroborated by prior research showing that those who have experienced sexual and physical abuse have higher dissociative symptoms in adulthood (Lipschitz et al., 1996; Favaro et al., 1998). In particular, physical and sexual abuse by family members have been correlated with increased dissociative symptoms (Chu and Dill, 1990; Plattner et al., 2003). Even in cases of violence and abuse by family members who trust and rely on them (i.e., betrayal trauma), dissociation may have been involved as a potential mechanism that blocks trauma-related information to maintain necessary attachments. Thus, dissociation is conceptualized as an adaptive process aimed to maintain self-preservation and serve as protection against psychological distress. In addition, Chu and DePrince (2006) reported that rejective and inconsistent parenting due to the personal traumatic experience of betrayal by caregivers negatively influenced the ability of children to learn state regulation and increased the risk for developing dissociation. Thus, both caregivers’ and children’s betrayal trauma history have been thought to pay a critical role in the development of children’s dissociation (Fung et al., 2023).

Furthermore, according to previous studies, the inability of children to feel safe and secure, the lack of understanding and feedback from caregivers, and the absence of healing in times of distress may provoke insecure and disorganized attachments and may be predictors of dissociation (Lyons Ruth, 2003; Hesse and Main, 2006; Schimmenti and Caretti, 2016). These findings suggest that antecedents of dissociation are not limited to abuse experiences, which underscores the importance of parent–child dynamics. Recent studies indicate that, in childhood, overprotection/overcontrol by caregivers is critical to adversity associated with traumatic events, depressive dissociation (Şar et al., 2021; Şar and Türk-Kurtça, 2021; Wu et al., 2022), multiple psychological problems/illnesses (Herbert and Dahlquist, 2008), and other related problems. Experiences of overprotection and excessive control may interfere with the independent thinking and feeling of children, which leads to decreased self-esteem and self-efficacy and may contribute to learned apathy (Seedat et al., 2003). Overprotection also inhibits the acquisition of social skills (Parker et al., 1979; Bögels et al., 2001). Contrary to the background of these effects and considering the association between dissociation and inadequate control and immature social skills to cope with stress, it can be indicated that overprotection may be an environmental factor of dissociation. A link between overprotection and dissociation has also been suggested by studies reporting that dissociative experiences occur more frequently in individuals who have difficulty identifying their feelings due to overprotection (Mason et al., 2005). On the other hand, Modestin et al. (2002), while finding a positive correlation between overprotection and dissociation, noted that lack of care is a more substantial contributor to dissociation than overprotection. Furthermore, different authors have shown contradictory results regarding the effects of lack of care and overprotection on dissociation in target samples with and without psychiatric disorders and with different psychiatric symptoms, and no consistent findings have been obtained. Considering these limitations, further research is needed to confirm the effects of overprotection and lack of care as nurturing styles on dissociation in the Japanese population.

Traditionally, the relationship between attachment style and dissociation has often been attributed to an inappropriate nurturing environment. For example, numerous findings indicated that an attachment style which is classified as disorganized and undirected (Main and Solomon, 1986) due to an abusive nurturing environment forms the basis of dissociative disorders (Liotti et al., 2005). On the other hand, due to limited evidence, it is difficult to confirm the impact of attachment styles with a background of non-abusive nurturing styles on dissociation. Schore (2001), who described the importance of a receptive nurturing style, stated that children control discomfort based on a sense of accepted security and safety wherein brain function for emotional regulation is developed. In this process, stable attachments are formed, but the lack of emotional reactivity of the caregiver can be traumatic (Dutra et al., 2009). In other words, it can be concluded that the reception of negative emotions is deeply related to attachment formation. Moreover, communication that denies negative emotions (discomfort and anxiety) hurts negative emotion regulation (Davidov and Grusec, 2006) and self-regulation of stress as well as the development of attachment security (Bugental, 2000). It can be speculated that dissociation may be frequently used to disassociate negative emotions from their negative impact on the (negative) self (Davidov and Grusec, 2006). Furthermore, dissociation may be enhanced by attachment styles formed against a background of nurturing styles, such as negative communication, but further research is needed to confirm this. Therefore, the aim of this study is to determine whether the effects of “lack of care” and “overprotection” as non-abusive nurturing styles on dissociation vary with attachment style.

### 1.2. Objective of the study

The present study first attempted to clarify the distribution of the DES scores in the general Japanese population. The relationship between the DES scores and mental health was also investigated in order to confirm whether the development of dissociation may harm the individual’s mental health. Furthermore, we focused on overprotection and lack of care as non-abusive nurturing styles and avoidance of intimacy and abandonment anxiety as attachment styles. We also examined their predictive influence on dissociation, respectively, and the role of attachment style in the association between nurturing styles and dissociation.

The hypotheses of this study are as follows:

*H1*: DES scores vary by age.

*H2*: DES scores vary by gender.

*H3*: DES scores correlate with the level of mental health, and higher DES scores are associated with poorer mental health.

*H4*: Nurturance style and attachment style contribute to dissociation.

*H5*: The impact of nurturing style on dissociation varies by attachment style.

## 2. Methodology

### 2.1. Target population

From March to April 2020, with the help of an Internet research company (Macromill, Inc.), we surveyed the registered collaborators who were asked to complete the study questionnaires. A certain age and developmental level are necessary to understand the content of the DES. Thus, we decided to conduct the survey among individuals aged 18 years or older. Moreover, to ensure that the survey’s targeted population was representative of the general population of Japan, residents aged 18–69 in 47 prefectures in Japan were selected. The number of participants by gender and age was adjusted to the Statistics Bureau of Japan (2015) (Table 1).

**Table 1:** Comparison of sample descriptive statistics to census data for study.

Age	Characteristics of the study sample				Census (2015)			Thousand people
	Population (%)	Female (%)	Male (%)	M/F	Population (%)	Female (%)	Male (%)	M/F
18–19	32 (3.1)	15 (1.4)	17 (1.6)	1.13	6,078 (7.1)	2,993 (7.0)	3,085 (7.2)	1.03
20–29	155 (14.9)	77 (7.4)	78 (7.5)	1.01	12,378 (14.4)	6,076 (14.1)	6,302 (14.6)	1.04
30–39	197 (18.9)	98 (9.4)	99 (9.5)	1.01	15,607 (18.1)	7,718 (18.0)	7,889 (18.3)	1.02
40–49	233 (22.4)	116 (11.1)	117 (11.2)	1.01	18,395 (21.4)	9,126 (21.2)	9,269 (21.5)	1.02
50–59	195 (18.7)	98 (9.4)	97 (9.3)	0.99	15,445 (18.0)	7,748 (18.0)	7,698 (17.9)	0.99
60–69	230 (22.1)	118 (11.3)	112 (10.7)	0.95	18,099 (21.0)	9,288 (21.6)	8,811 (20.5)	0.95

The classification of the developmental stages based on age differs according to the developmental theories of various families and is not unified, while the boundaries of the age groups to be classified are ambiguous. Therefore, in this study, data was presented in 10-year age increments in accordance with the Ministry of Health, Labour and Welfare (2019) to provide essential data for DES scores rather than age classifications by developmental stage. The participants were categorized as 18–19, 20–29, 30–39, 40–49, 50–59, and 60–69 years of age, and a dummy variable based on 18–19 years of age was used in the analyses described below. The number of participants by gender and age was adjusted to the Statistics Bureau of Japan (2015) (Table 1).

### 2.2. Ethical considerations

This survey was approved by the Ethics Review Board of the [blinded for review]. The survey participants were briefed of the purpose of the study before agreeing to participate in the study. They were informed that the survey was anonymous and the information provided by them would not be used for any purpose other than research.

## 3. Materials and methods

### 3.1. Survey tools

Participants were asked to provide their age, gender, profession, history of psychiatric consultation, and current medications.

### 3.2. Dissociative experiences scale

The DES (Bernstein and Putnam, 1986) is a 28-item scale that measures dissociative experiences. Subsequently, Carlson and Putnam (1993) developed the DES-II, which is a simpler version of the DES. This study used the Japanese version of the DES-II translated by Tanabe and Ogawa (1992). The participants were asked how often they experienced each item in their daily lives (frequency of experience). The frequency of experience was measured using an 11-point scale ranging from 0 to 100%, with 10% intervals. The average score of all 28 items was calculated and used as the DES score. The DES-II includes aspects of pathological dissociation (DES-Taxon; eight items), such as symptoms of loss of control over normal psychosomatic functions (e.g., amnesia, stupor, and conversion symptoms), and pathological tendencies such as unintentional and significant changes in consciousness and behavior (identity disorder). It also includes 20 items for average dissociation (Normal Dissociative Index), which are mild and temporary. Scores for pathological (DES-Taxon) and average dissociation (DES- Normal Dissociative Index) were calculated as the mean of corresponding item scores. The reliability and validity of this scale have been statistically validated, and it is the most commonly used dissociation questionnaire in most studies (Tanabe and Ogawa, 1992).

### 3.3. Japanese version of the WHO-5

The WHO-5-J is the Japanese version of the WHO-5 as developed by the World Health Organization (WHO). The standardization work for this index was completed in 2007 (Awata et al., 2007), and its reliability and validity have been confirmed. The scale consists of five items and determines the individual’s mood over the last 2 weeks. Answers were requested using the six-case method, ranging from “never” to “always.” Scores can range from 0 to 25, wherein a higher score indicates better mental health. Scores less than 13 indicate poor mental health, and a test for depression based on the International Classification of Diseases (ICD)-10 is necessary (Awata et al., 2007).

### 3.4. Parental bonding instrument

The PBI was developed by Parker (1984). It consists of two factors: a nurturance factor, which measures the degree of parental affection and attachment, and an overprotection factor, which measures the degree of parental overprotection and overinterference (Ogawa, 1991). Answers were requested using the four-case method, ranging from “not at all” to “very much so.”

### 3.5. Experiences in close relationships inventory

Brennan et al. (1998) used the Internal Working Model as a model for the Experiences in Close Relationships inventory (ECR scale). Based on this, Nakao and Kato (2004) created the ECR-GO (Experiences in Close Relationships inventory-generalized-other-version) as a scale to measure attachment styles toward others in general, which consists of 36 items and two dimensions of “avoidance of closeness” and “abandonment anxiety.” Ratings were obtained using a 7-point scale ranging from “very often true” to “not at all true.”

### 3.6. Data analysis

A statistical significance level of 0.05 was considered statistically significant, and all analyses were conducted using SPSS 25 for Mac.

## 4. Results

### 4.1. Demographic attributes of the participants

A total of 1,044 questionnaires were collected. The final number of participants included in the analysis was 1,042 (a valid response rate of 99.8%). Moreover, two participants with missing data for the DES, WHO-5-J, age, gender, and mental status were excluded.

Table 1 shows the distribution of the number of individuals in each age group and the ratio of men to women, along with the corresponding data from the 2015 Population Census. In addition to age, gender, and region, we also considered occupational bias in the demographics of the target population.

### 4.2. Descriptive statistics of DES scores

The mean and standard deviation (hereinafter referred to as *SD*) of the DES scores were 7.3 ± 13.7, with a median of 1.8. The mean scores of each item ranged from 4.7 (Item 4) to 14.1 (Item 2).

**Figure 1:**
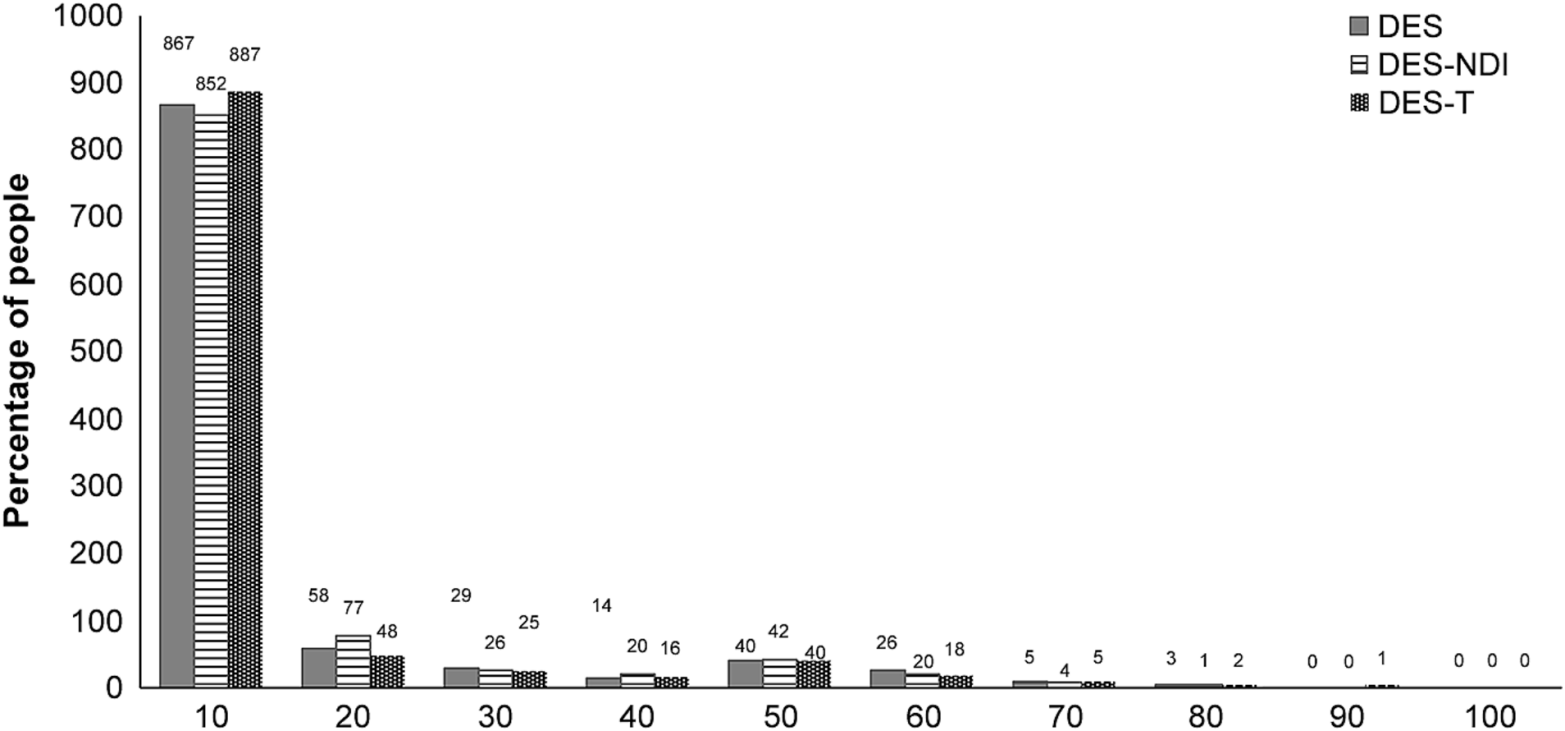
Distribution of the Dissociative Experience Scale (DES) scores.

The mean DES scores for the six age groups (10 years) were as follows: 18–19 years, 15.5; 20–29 years, 14; 30–39 years, 8.9; 40–49 years, 7; 50–59 years, 3.9; and 60–69 years, 3.4. Figure 1 shows the distribution of the DES scores. Of the total number of participants (520 men and 522 women), 954 (91.5%) had a score less than 30, while 88 (8.5%) had a score of 30 or higher. Thus, the distribution of the DES scores was skewed toward the lower range and did not follow a normal distribution (*p* < 0.05).

### 4.3. Gender differences

The Mann–Whitney *U* test indicated a significant difference in the DES scores based on gender (*Z* = −2.9, *p* = 0.003).

### 4.4. Relationship between DES and well-being

Spearman’s correlation coefficients between each scale were determined to determine the relationship between well-being and the DES score, pathological dissociation score, and average dissociation score. The results showed that Spearman’s ρ values were − 0.26 (*p* < 0.01), −0.23 (*p* < 0.01), and − 0.26 (*p* < 0.01), respectively (Table 2).

**Table 2:** Descriptive statistics of the sample and correlations between measures.

Scale	M	SD	DES	DES-NDI	DES-T	Well-being	Avoidance of intimacy	Abandonment anxiety	Care	Over protection
DES	7.27	13.71	1	0.99**	0.82**	−0.26**	0.11**	0.25**	−0.22**	0.26**
DES-NDI	7.59	13.30	0.99**	1	0.76**	−0.25**	0.09**	0.24**	−0.21**	0.25**
DES-T	6.21	13.72	0.82**	0.76**	1	−0.23**	0.17**	0.21**	−0.26**	0.26**
Well-being	12.42	5.73	−0.26**	−0.25**	−0.23**	1	−0.17**	−0.22**	0.30**	−0.28**
ECR-GO										
Avoidance of intimacy	61.74	14.55	0.11**	0.09**	0.17**	−0.17**	1	0.21**	−0.27**	0.21**
Abandonment anxiety	32.39	9.39	0.25**	0.24**	0.21**	−0.22**	0.21**	1	−0.23**	0.27**
PBI										
Care	21.97	7.52	−0.22**	−0.21**	−0.26**	0.30**	−0.27**	−0.23**	1	−0.66**
Overprotection	14.63	6.31	0.26**	0.25**	0.26**	−0.28**	0.21**	0.27**	−0.66**	1

^*^p < 0.05; ^**^p < 0.01; ^***^p < 0.001.

DES, Dissociative Experience Scale; DES-NDI, Normal Dissociative Index; DES-T, DES-Taxon; PBI, Parental Bonding Instrument; ECR-GO, Experiences in close relationships inventory-the-generalized-other-version.

### 4.5. The effects of attachment and nurturing styles on dissociation

The effects of the PBI subscale (care, overprotection) and ECR-GO subscale (avoidance of intimacy, abandonment anxiety) and their respective interactions on the DES scores were examined. Hierarchical multiple regression analysis was conducted with DES-Normal Dissociation Index (DES-NDI) scores and DES-Taxon (DES-T) scores as dependent variables. In Step 1, the dummy variables for the gender and age categories were used as control variables. In Step 2, the PBI subscale scores and ECR-GO subscale scores were entered to examine the main effects. In Step 3, the interaction terms for the PBI and ECR-GO subscale scores (with each variable converted to a centralizing variable and then multiplied together) were included.

First, hierarchical multiple regression analysis with DES-NDI scores as the dependent variables showed that the regression equation for Step 1 was significant [*F* (6,1,035) =19.35, *p* < 0.001] and the main effects of gender and age were significant. For Step 2, ΔR2 was significant [ΔR2 = 0.07, *F* (10,1,031) = 20.53, *p* < 0.01]. Moreover, males also showed higher DES-NDI scores than females (*β* = 0.12, *p* < 0.001). Compared with the 18- to 19-year-old age group, the 30–69-year-old age group showed lower DES-NDI scores with increasing age (*β* = −0.23, −0.30, −0.36, −0.38, *p* < 0 0.001). Furthermore, only the main effects of CA (*β* = −0.12, *p* = 0.002) and OP (*β* = 0.12, p = 0.002) were significant as the PBI subscales. In Step 3, since ΔR2 was not significant [ΔR2 = 0.004, *F* (14,1,027) = 15.07, *p* < 0.001], the model from Step 2 was finally selected (Table 3).

**Table 3:** Hierarchical multiple regression analysis with DES-NDI and DES-T as the dependent variables.

Scale	DES-NDI			DES-T		
	Standardized coefficients *β*			Standardized coefficients *β*		
	Step1	Step2	Step3	Step1	Step2	Step3
SEX	0.14***	0.12***	0.12***	0.14***	0.12***	0.13***
Age dummy 1	−0.05	−0.06	−0.06	−0.02	−0.02	−0.02
Age dummy 2	−0.19**	−0.23**	−0.22**	−0.17*	−0.19**	−0.19**
Age dummy 3	−0.27***	−0.30***	−0.30***	−0.23**	−0.25**	−0.25***
Age dummy 4	−0.34***	−0.36***	−0.36***	−0.30***	−0.31***	−0.31***
Age dummy 5	−0.38***	−0.38***	−0.38***	−0.32***	−0.32***	−0.32***
ECR-GO						
Avoidance of intimacy		0.05	0.05		0.10**	0.10**
Abandonment anxiety		0.06	0.06		0.03	0.03
PBI						
Care		−0.12**	−0.11**		−0.09**	−0.09*
Overprotection		0.12**	0.13**		0.12**	0.13**
Care × avoidance of intimacy			0.03			0.03
Overprotection × avoidance of intimacy			0.06			0.06
Abandonment anxiety × CA			−0.004			−0.004
Abandonment anxiety × OP			0.03			0.04
*F*	19.35***	20.53***	15.07***	24.73***	21.85***	14.98***
R2	0.10	0.17	0.17	0.09	0.15	0.15
Adjusted R-squared	0.10	0.16	0.16	0.08	0.14	0.14
ΔR2	0.10***	0.07***	0.004	0.09***	0.06***	0.004

*p < 0.05; **p < 0.01; ***p < 0.001.

DES, Dissociative Experience Scale; DES-NDI, Normal Dissociative Index; DES-T, DES-taxon; PBI, Parental Bonding Instrument; ECR-GO, Experiences in close relationships inventory-the-generalized-other-version.

Next, in a hierarchical multiple regression analysis with the DES-Taxon score as the dependent variable, the regression equation for Step 1 was significant [*F* (6,1,035) = 16.48, *p* < 0.001], as were the main effects of gender and age. For Step 2, ΔR2 was significant [ΔR2 = 0.06, *F* (10, 1,031) = 17.57, *p* < 0.001]. Moreover, the males also showed higher DES-NDI scores than the females (*β* = 0.12, *p* < 0.001). Compared with the 18–19-year-old group, the 30–69-year-old group showed lower DES-NDI scores with increasing age (*β* = −0.19, −0.25, −0.31, −0.32, *p* < 0 0.001). Furthermore, only the main effects of CA (*β* = −0.09, *p* = 0.018) and OP (*β* = 0.12, *p* = 0.003) were significant as the PBI subscales. In Step 3, since the ΔR2 was not significant [ΔR2 = 0.004, F (14,1,027) = 12.93, *p* < 0.001], the model in Step 2 was finally selected (Table 3).

## 5. Discussion

This study provided the necessary data on the distribution of dissociative experiences in the general Japanese population with an age range of 18 to 69 years (Statistics Bureau of Japan, 2015). Furthermore, the effects of gender, age, nurturing style as an environmental factor and attachment style as an individual factor on dissociative experiences were investigated. The results were then partially consistent with the research hypotheses.

### 5.1. Distribution of the DES scores in the general population

The DES score for the sample in this study was 7.27. In studies from other countries, the frequency of dissociative experiences was investigated in the general population. In Sivas, Turkey, the mean DES score of 994 people (women, 47.2%; men, 52.8%; mean age, 43.7 ± 12.9) was 6.7 (±6.1; Akyüz et al., 1999). In Winnipeg, Canada, the mean DES score for 1,055 people (women, 58.3%; men, 41.7%; mean age, 43.2 ± 16.7) was 10.8 (±10.1; Ross et al., 1990), indicating that DES scores differ by country. In Shanghai, China, Xiao et al. (2006) first reported that the average score on DES was 2.6 for 618 factory workers, 4.1 for 423 psychiatric inpatients, and 4.5 for outpatients, consistent with the rate of physical and sexual abuse in childhood. More recent studies in Hong Kong and Taiwan demonstrate that the prevalence of pathological dissociation varies in the Chinese population, ranging from 0.3 to 4.5% in nonpsychiatric settings and 1.7–19.5% in psychiatric settings (Chiu et al., 2017; Fung et al., 2021). These studies also support the association between pathological dissociation and cumulative trauma experiences (Chiu et al., 2015, 2017; Fung et al., 2018).

Furthermore, the mean DES scores for each age group were examined and showed that the 18–19-year-old and 20–29-year-old groups had the highest DES scores, while the 60–69-year-old groups had the lowest DES scores. These results are consistent with the findings of previous studies (Putnam, 1993). Therefore, if a DES score is higher among those aged 60–69 years, it would be a rare case, and a detailed assessment of mental health risk is necessary after confirming that there is no misunderstanding of the content of the DES.

### 5.2. Frequency of dissociative experiences and association with well-being

The present study suggests a weak relationship between dissociative experiences, DES-NDI, DES-T, and well-being in the Japanese population. The WHO-5-J, which was used for the self-assessment of mental health, measures an individual’s general well-being and is often used to screen for depression. A score of 13 or less on this scale indicates poor mental health. Furthermore, WHO-5 scores are significantly negatively correlated with Short General Health Questionnaire scores (GHQ-12, where higher scores indicate poorer mental health, stress, and suicidal ideation), Patient Health Questionnaire-9 scores (*r* = −0.52), Hospital Anxiety and Depression Scale-anxiety (HADS; *r* = −0.524), and HADS-depression (*r* = −0.63; Ramkisson et al., 2016). In other words, people with a high frequency of dissociative experiences and low WHO-5 scores are more likely to experience stress and mental illness.

### 5.3. Association of dissociative experiences with nurturing and attachment styles

This study examined the factors influencing dissociative experiences regarding gender, age, nurturing attitudes, and attachment styles. The relative importance of each factor in increasing dissociative experiences was also determined. The results indicated that gender, age, and “care” and “overprotection” as nurturing attitudes are contributing factors to dissociation (DES-NDI and DES-T). Considering that inadequate nurturing is associated with nonfunctional stress coping and depression (Galambos et al., 2003), overprotection and inadequate care as nurturing styles may have habituated aversive stress coping and influenced the nonfunctional defense mechanism of dissociation. Moreover, “avoidance of intimacy” as an attachment style contributed to pathological dissociation. On the other hand, the impact of “care” and “overprotection” on average and pathological dissociation was not influenced by attachment style, which is contrary to the hypothesis. In other words, it is interesting to note that regardless of the degree of attachment style, the impact of nurturing styles, such as overprotection and inadequate care on dissociation cannot be underestimated.

Şar and Türk-Kurtça (2021) also reported that harsh parental criticism and overprotection/overinterference may foster dissociation mediated by vulnerable narcissism. Moreover, Linde-Krieger et al. (2022) found that intrusive and autonomy-impairing child nurturing behaviors predict high levels of dissociation at age of 5 years. Thus, the parenting styles described by people with dissociative disorders appear to be consistent. In line with the findings of previous research, the present study suggests that overprotection may also be a risk factor for dissociation in the general population.

According to Offen et al. (2003), overprotection is associated with depressive symptoms via dissociation, but when the mediating effect of dissociation is removed, the association between overprotection and depression disappears. In other words, the presence or absence of dissociative symptoms due to overprotection is suggested to be an essential factor in whether or not depression is induced. This study indicates that overprotection is a contributing factor to dissociation and supports the possibility that depression may concurrently occur with dissociation where overprotection is present in the background, suggesting that mental health problems should be considered. Kawabata et al. (2011) also distinguished “over-involvement” from child-centered, “watchful” behavior, which is caused by parents’ feelings of anxiety and psychological urge to keep their children under control. In order to ascertain the influence of the controlling psychology behind intrusive and inconsistent behavioral traits on the increase in dissociative experiences, it may be necessary for the future to focus on the presence or absence of a controlling psychology in addition to the behavioral traits of the caregiving style.

Considering the sociocultural perspective on the present results, the decline in the fertility rate in East Asia including Japan (Matsuda, 2020) as well as China with one-child policy may constitute a social context for overprotection as a parenting style. For example, the nuclear family may cause parents to focus their attention on a small number of children and to overprotect and overinterfere with them, leaving children vulnerable to stressful life events as they grow up. Thus, future comparative studies need to explore the relationship between overprotection and dissociation across cultures, not only in East Asia but also in other areas.

Care as a nurturing style implies acceptance and support. Positive acceptance is essential in developing the child’s self-concept and inhibits avoidant behavior (Flett et al., 1995). It has also been reported that decisive care is associated with resilience and may alleviate stress and mood disorders (Jones et al., 2000). On the other hand, if there are fewer supportive and accepting experiences, children have fewer opportunities to learn functional coping skills through interactions with others. This may constitute a background that favors avoidant coping strategies for realistic difficulties and may contribute to dissociation.

Kate et al. (2023) argue that one’s ability to seek care and comfort from parents and to develop self-esteem and self-worth may be a protective factor for dissociation as a part of positive parent–child dynamics. In contrast, overprotection/overinterference and the lack of care as negative nurturing attitudes may interfere with the safety experiences of children and contribute to apathy and dissociation (Schimmenti and Caretti, 2016). Thus, the present study suggests the possibility that mental disorders associated with nurturing styles, such as lack of care and overprotection, may be exacerbated by the frequency of dissociation, which requires future research on dissociation and nurturing. Usual dissociation is distinguished from pathological dissociation in that it is understood as not interfering with daily life. However, it has been reported that individuals with dissociative tendencies exhibit more fragmented aversive memories and anxiety than those who do not (Kindt and van den Hout, 2003). Moreover, dissociation as a negative coping strategy may interfere with developing effective cognitive strategies and increase the risk of developing depression (Offen et al., 2003). Therefore, the frequency of usual dissociation with aspects of aversive coping should be considered.

Furthermore, this study found that avoidance of intimacy as an attachment style influences pathological dissociative symptoms. Intimacy avoidance reflects expectations of others and negative beliefs about others, which can be determined by the following question: “Can others help me or can I trust them?” (Bartholomew and Horowitz, 1991). It has been reported that people with high levels of intimacy are stressed in their relationships with others and are refusing to open up to others and that high levels of intimacy avoidance tend to promote negative cognitions and behaviors in interpersonal situations (Collins and Feeney, 2004). In other words, difficulties in accessing positive information and less experience in coping with stress by using the support of others are associated with avoidant stress coping (Simpson et al., 1992), which may exacerbate dissociative symptoms.

On the other hand, the results showed that the effects of nurturing styles, such as overprotection and inadequate care, on dissociation did not change depending on the attachment style. This result indicates the importance of nurturing style in increasing dissociation. In other words, non-abusive nurturing styles should be considered, as they may also contribute to dissociation by forming aversive coping strategies and vulnerability to negative emotions. In this regard, mental health professionals and educators who support both mothers and children need to note the fact that, due to overprotection by mothers, children’s perception to feel helpless and out of control over their lives, and being isolated from the outside world may be associated with the development of dissociation. This is especially important in East Asian countries, where the general people are insensitive to the adverse effects of overprotection on children (Wu et al., 2022). In addition, parental support and positive feedback may lead to the development of resilience to adversity and the ability to process and recover from traumatic experiences (Kate et al., 2023). However, to further confirm the results of the present study, further research is expected to focus on the psychological aspects behind negative emotion control, social community and social skills, and nurturing behaviors and examine their relationship with dissociation.

## 6. Conclusion

This study determined the distribution of the frequency of dissociative experiences in the general Japanese population and its association with an individual’s well-being. We also suggested that overprotection and lack of care as nurturing attitudes may constitute a background for preferentially choosing avoidant coping strategies for realistic difficulties and promote frequent use of dissociation. Considering that the frequency of dissociative experiences may exacerbate mental illness, this study presents essential data for timely intervention and supportive-preventive efforts regarding dissociative experiences. However, further research is needed to confirm whether deficits in emotional regulation and social skills are behind the demonstrated impact of intimacy avoidance as an attachment trait on pathological dissociative symptoms. Despite such limitations, the findings of this study would still significantly contribute to the body of knowledge on dissociative analog studies that examine aspects of the developmental environment and individual characteristics formed by it.

The present study was conducted in the general population of Japan, and the association between pathological dissociation and well-being was low. Further research is needed to determine the clinical relevance of discussing mental health risk associated with pathological dissociation. The DES is widely used as a scale that has been validated for reliability and validity. According to Tanabe (2004), investigators frequently use a cutoff score of 30 to determine the likelihood of pathological dissociation, while the cutoff score rarely exceeds 20 in Japanese individuals with dissociation. As a limitation of this study, however, the criteria for a cutoff score of DES in the general Japanese population have not been examined and await further research.

Factual findings will require follow-up studies that include comprehensive mental health functioning assessments and structured diagnostic interviews for those with high DES scores. Another limitation of this study is that the actual child-rearing environment is not transparent since the PBI was assessed with a self-administered questionnaire. Future long-term longitudinal studies that consider the relationship between caregivers and children’s perceived caregiving attitudes and gender differences among caregivers will be required.

## Data availability statement

The raw data supporting the conclusions of this article will be made available by the authors, without undue reservation.

## Ethics statement

The studies involving human participants were reviewed and approved by the Research Ethics Committee, Department of Clinical Psychology, Kyushu University Graduate School of Human-Environment Studies. The studies were conducted in accordance with the local legislation and institutional requirements. The participants provided their written informed consent to participate in this study.

## Author contributions

BW and TK contributed to the conception and design of the study. BW organized the database and performed the statistical analysis and wrote the first draft of the manuscript. TK wrote sections of the manuscript and advised revising the manuscript. All authors contributed to manuscript revision and read and approved the submitted version.

## Funding

This work was supported by Japan Society for the Promotion of Science (JSPS) Grants-in-Aid for Scientific Research (KAKENHI) Grant Number JP21K03087.

## Conflict of interest

The authors declare that the research was conducted in the absence of any commercial or financial relationships that could be construed as a potential conflict of interest.

## Publisher’s note

All claims expressed in this article are solely those of the authors and do not necessarily represent those of their affiliated organizations, or those of the publisher, the editors and the reviewers. Any product that may be evaluated in this article, or claim that may be made by its manufacturer, is not guaranteed or endorsed by the publisher.
